# The puzzle of scientific progress: cultivating problem solvers

**DOI:** 10.3389/fpsyg.2026.1732871

**Published:** 2026-07-23

**Authors:** Mariana Araújo-Pereira, Bruno B. Andrade

**Affiliations:** 1 Laboratory of Clinical and Translational Research, Gonçalo Moniz Institute, Oswaldo Cruz Foundation, Salvador, Brazil; 2 The Sybil Lab, MONSTER Institute for Education, Care, Research, and Technological Development in Health, Salvador, Brazil; 3 Division of Infectious Diseases, Department of Medicine, Johns Hopkins University, Baltimore, MD, United States; 4 Department of International Health, Bloomberg School of Public Health, Johns Hopkins University, Baltimore, MD, United States; 5 The Bright Lab, MONSTER Institute for Education, Care, Research, and Technological Development in Health, Salvador, Brazil

**Keywords:** education, problem solver, psychology, science, scientific progress

## Abstract

Human nature is paradoxical. People often recognize problems but hesitate to act, preferring the familiarity of discomfort to the uncertainty of change. This reluctance to embrace the unknown is not just a personal tendency; it extends to science and society at large, where progress is often stalled by fear of disruption. To overcome stagnation, it is increasingly important to educate and develop problem solvers, or in other words, dissolvers of chaos—those who transform disorder into understanding, uncertainty into insight. This conceptual commentary explores the mindset of problem solvers, examining how they approach complexity not as an obstacle, but as a puzzle to be solved. Just as assembling a jigsaw puzzle requires patience, adaptability, and the ability to identify connections, solving real-world problems demands a willingness to navigate uncertainty, persist through failure, and engage in deep, iterative thinking. We examine historical and anecdotal examples of resistance to change, discuss the cognitive and social barriers that discourage deep problem-solving, and propose strategies for fostering a new generation of problem solvers. By shifting perspective, from seeing problems as obstacles to recognizing them as opportunities to build new knowledge, bit by bit, we can cultivate individuals who not only navigate complexity but embrace it as the foundation of progress.

## 1 Introduction

There is something curious about human behavior: people often prefer to stay in uncomfortable situations, even when they know better alternatives exist. They complain about what bothers them, acknowledge that they are stuck in a problem, yet when it comes time to act, they hesitate. This occurs because the new is frightening. Uncertainty is unstable ground, and no matter how difficult the current reality may be, it is still familiar. It is the paradox of those who suffer yet fear change (Tversky and Kahneman, 1979).

This paralysis in the face of the unknown is not limited to personal decisions—it is also present in science and academia. In a world where knowledge advances rapidly, we cannot afford to cling to what we already know simply because it is comfortable. Science needs people who have the courage to take the first step, to endure the initial discomfort in search of solutions. But true problem solvers do more than just move forward: they embrace mistakes, disagreement, and discordance as essential parts of the process. It is through these challenges that out-of-the-box solutions emerge, pushing science beyond conventional limits (Samuelson and Zeckhauser, 1988). They are those who begin to assemble a puzzle without knowing the image it will reveal, willing to bring order to scattered pieces. In doing so, they act as dissolvers of chaos, transforming confusion into meaning. In an increasingly complex and rapidly changing world, such individuals are needed now more than ever.

## 2 Science needs those who are not afraid of problems

Building on this paradox, the problem is not just the fear of the new but the inability to project the future and understand that today’s discomfort may be necessary for something far better tomorrow. This happens because our brains disproportionately value the present over the future: a phenomenon known as hyperbolic discounting (Winkler, 2006). This cognitive bias leads individuals to favor immediate certainty and comfort over potentially more beneficial, but uncertain, future outcomes. Consequently, in the short term, effort feels large and waiting feels costly; over longer horizons, perspective often changes.

This same logic influences how people deal with problems. Science is full of complex questions, yet many prefer to stick with what has already been done, what is already established, rather than face the initial discomfort of questioning, testing, and innovating. Searching for new answers can seem exhausting, difficult, risky, yet it is precisely what drives knowledge forward and changes lives. The initial discomfort of change is inevitable. And this is precisely where many people give up. They look at the effort required and think, “This is going to be too hard,” without realizing that this initial effort is only a fraction of the journey. If they projected the future clearly, they would see that taking the first step is always the hardest part—and that after that, movement becomes easier, more natural, more automatic. As Newton’s first law states, an object at rest will remain at rest unless acted upon by an external force, but once it is set in motion, it is easier to continue in that state (Cohen and Smith, 2002). People and institutions often resist intellectual and scientific change. It takes a deliberate force (whether in the form of a disruptive thinker, groundbreaking evidence, or an urgent social need) to overcome this resistance and propel science forward.

How many advancements were delayed by resistance to the new? How many discoveries took years longer than necessary because people feared stepping out of their comfort zones? History offers countless examples of this tension between comfort and progress. When Ignac Semmelweis first proposed handwashing as a way to prevent infections in hospitals, his ideas were dismissed, until later research confirmed he was right, revolutionizing medical hygiene (Tyagi and Barwal, 2020). Barry Marshall’s claim that *Helicobacter pylori* causes ulcers met skepticism until his self-experiment catalyzed a paradigm shift in gastroenterology (Marshall and Adams, 2008). Even anesthesia initially faced opposition from those who saw pain as essential to healing, and the use of anesthesia was associated with unprofessional behavior and profit seeking (Meyer and Desai, 2015). These cases illustrate recurring patterns of systemic resistance and the necessity of acting despite fear.

## 3 Similar patterns can be observed across multiple scientific disciplines

John Snow’s proposal that cholera spread through contaminated water challenged dominant miasma theories and initially faced considerable resistance before becoming a cornerstone of modern epidemiology (Tulchinsky, 2018). Alfred Wegener’s theory of continental drift was also dismissed for decades before later geophysical evidence supported the foundations of plate tectonics (Greene, 1989). More recently, early warnings about climate change and antimicrobial resistance were frequently underestimated despite accumulating evidence. These examples demonstrate that resistance to disruptive ideas is not an isolated phenomenon, but rather a recurring characteristic of human and institutional behavior in the face of uncertainty and paradigm change. Progress rarely arrives because courage appears; it begins when someone challenges convention and persists. The academic system and barrier to problem solvers.

Scientific research is built on unanswered questions. Yet, too often, the academic system itself creates barriers for those who genuinely try to solve problems. The pressure to publish quickly, the resistance to new ideas, and bureaucratic hurdles make it easier to follow the safe path than to take risks. But science without problem-solving is not science: it is stagnation.

Although science thrives on innovation, it paradoxically enforces a status quo. Scientific journals often favor confirmatory studies over disruptive discoveries, and funding agencies reward productivity over boldness. The system prioritizes incremental advances (safe, predictable studies that ensure a steady stream of publications) over riskier, paradigm-shifting research that truly advances our understanding of natural phenomena. Thus, many researchers iterate on incremental ideas that pass review without materially advancing understanding (Park et al., 2023).

This is not just an academic inconvenience; it actively slows progress. Take, for instance, the development of mRNA vaccines. The foundational research behind this revolutionary technology was met with skepticism, underfunded, and dismissed for years. In 2005, Katalin Karikó and Drew Weissman’s paper demonstrating how nucleoside modifications could make mRNA therapeutically viable was rejected by top journals for being “not novel” and “not of broad interest.” It took over a decade for the scientific community to fully recognize its potential (Bansal, 2023). Had their findings been accepted and explored sooner, the medical world might have been better prepared for pandemics like COVID-19. Instead, resistance to novel ideas delayed one of the most significant advancements in modern medicine.

If the fear of the new and procrastination were merely personal issues, they would be individual problems. But when this happens in science, the impact is collective. The lack of problem solvers in academia directly affects the advancement of knowledge, the development of public policies, innovation in industry, and even how we handle global crises. What separates a scientist from someone who merely repeats information is precisely the willingness to face the unknown, to experiment, fail, recalibrate, and try again, despite the systemic barriers.

## 4 The decline of deep thinking and the illusion of knowledge

Even as access to information expands, appetite for intellectual risk appears to shrink. The modern environment rewards speed and surface understanding over depth. Education systems often prioritize standardized performance and efficient correctness rather than exploration and complexity. Students learn to deliver quick answers, not to sustain inquiry when answers are slow or uncertain. Standardized tests, rigid curricula, and performance-driven metrics reinforce a system where critical thinking takes a backseat to memorization and repetition. Instead of fostering curiosity, resilience, and independent inquiry, education often trains individuals to operate within established frameworks rather than challenge them.

The long-term consequences of this approach are clear. Research suggests that creativity and problem-solving abilities decline as children progress through formal education, and this has gotten worse in recent decades (Kim, 2011). Without the ability to tolerate failure and explore open-ended questions, students grow into professionals who prefer predictability over discovery (Kim, 2011). Sagan (1994) argued that when children are discouraged from exploring and asking questions, they are robbed of the tools necessary to shape their own future. A society that treats curiosity as an inconvenience rather than a virtue will inevitably struggle to foster future scientists, innovators, and problem solvers.

### 4.1 Educational models

There is evidence that alternative educational models can improve problem-solving abilities, from John Dewey’s to science, technology, engineering and mathematics (STEM) approaches that cultivate critical and creative thinking. Early in the twentieth century, Dewey’s progressive pedagogy promoted learning by doing and inquiry-driven education, positioning the classroom as a space for experimentation and critical thinking (Miettinen, 2000). Shortly after, George Polya’s (2014) heuristics provided practical strategies for tackling difficult problems. Jean Piaget deepened this perspective by situating problem-solving within the dynamics of cognitive development and social interaction (Börnert and Wilbert, 2015). Freire (1970) emphasized critical pedagogy, framing education itself as a process of confronting and resolving problems of oppression and inequity. Concomitantly, Lev Vygotsky’s sociocultural theory placed collaborative interaction at the center of learning (Vygotsky and Cole, 1978). Together, these perspectives highlight that problem-solving is not innate, and can be cultivated when education prioritizes reasoning, resilience, and social context.

Project-based learning and STEM curricula operationalize these ideas. A STEM program enhanced five-year-olds’ problem-solving skills with effects persisting even weeks after the program ended (Sahin, 2021). More recent evidence demonstrates that complex problem-solving skills can be intentionally cultivated across educational levels. A systematic review by Lu and Xie (2024) found that the deliberate integration of educational technologies into structured instructional strategies consistently improved students’ problem-solving abilities (Lu and Xie, 2024). Complementing, other study showed that higher education practices foster key dimensions of complex thinking, such as interdisciplinary knowledge integration and innovative solution generation (Ramírez-Montoya et al., 2025). Perhaps, the key to cultivating a new generation of problem solvers may lie in greater investment in early childhood, but also primary, secondary and higher education, with a curriculum that prioritizes STEM learning, the development of scientific and critical thinking, logical reasoning, resilience, and patience.

### 4.2 The culture of immediacy and techno-cognitive dependency

Another aspect that can mitigate the creation and stimulation of problem solvers is the culture of immediacy. In today’s digital age, everything is designed for speed and efficiency, communication, entertainment, and even learning. The internet, social media, and the constant acceleration of industry have created a culture in which people expect instant gratification in all areas of life. Information that once took years to master can now be accessed in seconds. But does this mean we are becoming more knowledgeable? Or does the illusion of knowledge make us less capable of critical thinking and problem-solving?

Information that once demanded years to master is now seconds away, especially through Artificial Intelligence (AI) assistance. Access is valuable, but overreliance on tools can weaken cognitive endurance. Effortful retrieval and struggle are essential for durable learning (Bjork and Bjork, 2011). When answers arrive too easily, the brain stores them shallowly, and connections between ideas remain thin. This creates a techno-cognitive dependency, in which individuals can function as long as the tools are available, but struggle when they must think critically on their own.

If people become accustomed to receiving solutions without effort, they lose the ability to tolerate intellectual discomfort. If everything is expected to be solved quickly, frustration arises when solutions take longer than anticipated. This can lead to intellectual fragility, a decreased tolerance for difficult problems and a lower willingness to persevere through challenges. A generation accustomed to instant rewards and minimal cognitive effort may struggle to cope with obstacles that require sustained thinking (Rong et al., 2022).

Evidence suggests automation of everyday cognition has reduced cognitive exertion. A meta-analysis reports a 10–15% decline in higher-order skills across 30 years (George et al., 2024). Creativity also appears to stagnate (Kim, 2011), consistent with findings in divergent thinking (the ability to generate multiple, original solutions to ill-defined problems) (Runco and Acar, 2012). Neuroscience highlights ideation and flexible thinking as central to discovery (Benedek and Fink, 2019). If these trends continue, societies may produce tool-adept individuals who struggle with independent problem-solving. Yet the same technologies that tempt us to outsource thinking can also deepen it, when we choose to use them as instruments of inquiry rather than shortcuts to answers. Used deliberately, the internet becomes a research commons and AI becomes a Socratic partner: they widen the search, press for alternatives, and surface analogies, while judgment, values, and context remain human. In practice, this means designing learning and research so that machines act as co-pilots, not captains. If next generations of students and educators learn AI literacy, prompt design for inquiry (not answers), documentation of reasoning, and ethical guardrails, human + machine teams will do what neither could alone: cultivate deeper understanding, faster iteration, and more resilient problem solvers.

## 5 How to cultivate problem solvers

**Figure 1:**
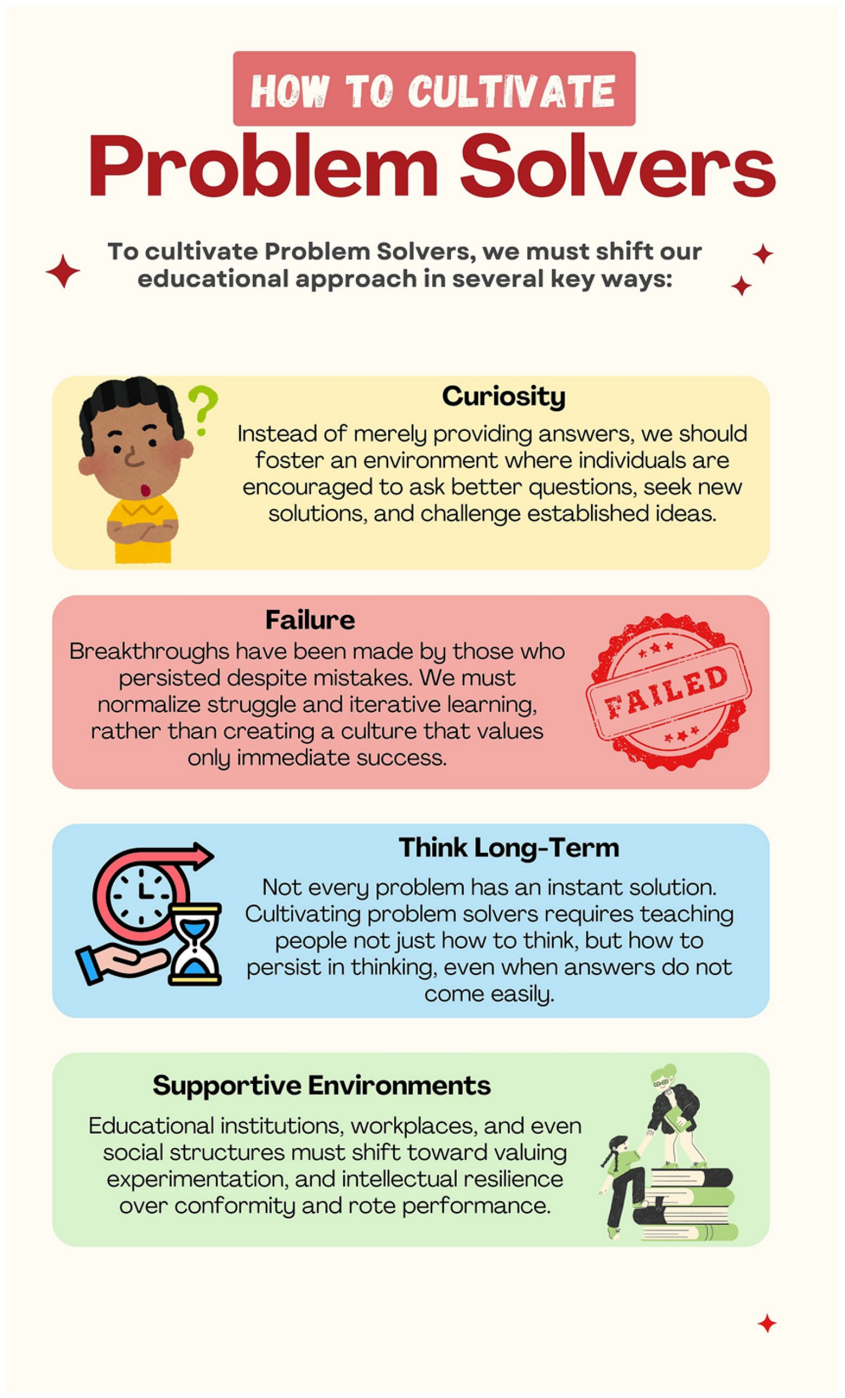
How to cultivate problem solvers. Illustration summarizing four essential pathways to foster effective problem solvers: curiosity, encouraging inquiry and critical questioning; failure, normalizing mistakes as part of iterative learning; long-term thinking, promoting persistence when solutions are not immediate; and supportive environments, creating educational and professional cultures that value experimentation and intellectual resilience over conformity.

The study of problem-solving has a long tradition in psychology and cognitive science. Early Gestalt theorists highlighted the role of insight in restructuring problems and discovering novel solutions (Wertheimer, 1938; Vitello and Salvi, 2023). Behaviorist perspectives, in contrast, emphasized trial-and-error learning as a mechanism of problem resolution formalized through the “law of effect” (Thorndike, 1947; Davidson and Sternberg, 2003). Later, the information-processing paradigm framed problem-solving as a systematic search through a problem space guided by algorithms and heuristics, and inspired the development of AI (Simon and Newell, 1971; Tversky and Kahneman, 1981). Later work introduced frameworks distinguishing well- and ill-defined problems (Simon, 1973) and complex vs. simple problem contexts (Funke, 2010). More recent work has focused on constructs such as cognitive flexibility (Martin and Rubin, 1995) and adaptability, which align closely with the capacities we describe here. Reversing declines in problem-solving and critical thinking requires cultural and instructional shifts that normalize uncertainty and sustained effort. Thus, we must shift our approach in several keyways, as described in Figure 1.

### 5.1 Four pathways to cultivation

1. *Curiosity:* instead of merely providing answers, we should foster an environment where individuals are encouraged to ask better questions, seek new solutions, and challenge established ideas (Antonioni and Valdano, 2025).
2. *Failure:* breakthroughs have been made by those who persisted despite mistakes. We must normalize struggle and iterative learning, rather than creating a culture that values only immediate success (Han, 2020).
3. *Think long-term:* not every problem has an instant solution. Cultivating problem solvers requires teaching people not just how to think, but how to persist in thinking, even when answers do not come easily.
4. *Supportive environments:* educational institutions, workplaces, and even social structures must shift toward valuing experimentation, and intellectual resilience over conformity and rote performance.

Expertise research underscores that problem-solving develops through deliberate practice. Experts differ from novices not just in quantity of knowledge but in how they structure and apply it, focusing on deep features rather than surface details (Ericsson, 2006). Becoming a problem solver is less about innate talent than disciplined habits: patience, adaptability, and willingness to engage complexity. With these, problems shift from obstacles to opportunities.

## 6 The puzzle of science and life

Thus, solving problems is like putting together a puzzle. At first, everything seems like chaos—a bunch of disconnected pieces that make no sense together. It is tempting to give up because looking at a challenge without knowing where to start is frustrating. But what happens when you persist? First, you find the edges, create a structure, and begin to shape the problem. Then, you fit the pieces together little by little, testing, failing, adjusting. And over time, what once seemed impossible begins to take form before your eyes. The image emerges, piece by piece, until the problem is solved. Every solution, whether academia or life, emerges from this process: finding the first connections, making gradual adjustments, failing without fear, and continuing until everything makes sense.

And do you know what happens when you finally complete a puzzle? The satisfaction is so great that you often feel like picking up another one. And another. And another. Each one bigger, with more complex designs. Until, suddenly, solving puzzles stops being a difficult task and becomes something stimulating, exciting, enjoyable. What once seemed like an overwhelming problem now feels like an engaging challenge. And that is how a problem solver is born. You stop viewing problems as unsolvable obstacles and start seeing them as opportunities to bring order to chaos. In science, in academia, and in life, this is the difference between those who remain paralyzed and those who move forward. Solving problems is not a burden—it is a process, a game, an art. And once you learn to play, you never want to stop.

At their core, problem solvers are not only puzzle builders but dissolvers of chaos: they transform disorder into meaning by integrating scattered elements into coherent structures. This capacity appears across life, from tweaking a recipe to reorganizing after upheaval. The act of becoming a problem solver is itself a recursive puzzle: each solved challenge adds a piece to one’s internal model (knowledge, experience, adaptability) enhancing capacity for the next.

## 7 The future of problem solving

Seen from this lens, problem-solving has historically been an interdisciplinary endeavor, with psychology (Martin and Rubin, 1995; Vitello and Salvi, 2023), education (Freire, 1970; Miettinen, 2000; Börnert and Wilbert, 2015), computer science (Simon and Newell, 1971), and sociology highlighting how social networks and systemic inequalities influence the capacity to generate and implement solutions (Apiti and Chukwuemerie, 2024). These perspectives converge to show that problem-solving is a shared human practice shaped by individuals, institutions, and technologies.

At the same time, the field has long grappled with the challenge of measuring and evaluating such a multifaceted capacity. Early Gestalt researchers relied on tasks like Duncker’s candle problem to capture moments of insight (Weisberg and Suls, 1973). Mid-twentieth-century psychologists adopted structured laboratory tasks such as the Tower of Hanoi (Kotovsky et al., 1985), and computational simulations (Gallopoulos et al., 1994). More recent assessments, including international studies such as Programme for International Student Assessment (PISA), have attempted to evaluate applied problem-solving in educational settings (Stadler et al., 2020). Yet across eras, creativity, adaptability, and collaboration have proven resistant to standardized measurement. Advances in cognitive neuroscience underscore both the promise and the limitations of current approaches.

Looking ahead, AI, quantum computing, and large-scale analytics will reshape problems and required skills. Climate change, pandemics, and geopolitical instability demand international, interdisciplinary solutions. Education must adapt with AI-powered adaptive environments and experiential platforms that tailor instruction to styles, nurturing resilience and creativity alongside technical competence. Ethical questions will accompany these shifts: equity of access, algorithmic consequences, and over-reliance on aids. The likely path is synergy: human depth and creativity combined with computational power to address challenges beyond either alone. Problem solvers will not be replaced but expanded, becoming co-creators in an era of complexity.

## 8 Concluding remarks

Ultimately, the discussion here is not just about science or academia but about how the fear of the new can be paralyzing in any area of life. But knowledge and progress exist because, at some point, someone chose to face discomfort and act. What science—and the world—needs is exactly this: people willing to look at a problem and say, “Okay, now how do we solve this?”

The metaphor of the puzzle captures this mindset. At first, every problem appears as chaos: scattered pieces that seem impossible to connect. Yet persistence, testing, and adjustment gradually reveal an image that was hidden all along. If you have ever completed a puzzle and felt the satisfaction of fitting the last piece, you know what it means to solve a problem. But imagine carrying that feeling into every aspect of your life. Imagine turning every challenge into a puzzle to be solved. Imagine looking at chaos and not feeling powerless, but excited. This is the mindset of true problem solvers. And these are the people who change the world.

## Data Availability

The original contributions presented in the study are included in the article/supplementary material, further inquiries can be directed to the corresponding authors.

## References

[ref1] Antonioni A. Valdano E. (2025). Complexity72h: training the next generation of researchers in complex systems. Commun. Phys. 8, 1–4. doi: 10.1038/s42005-025-01979-5, 37880705

[ref2] Apiti A. A. Chukwuemerie O. C. (2024). Adult educators’ perception and application of Paulo Freire problem-solving techniques in training of adult learners in Awka south local government area. J. Res. Adult Contin. Educ. 3, 18–32.

[ref3] Bansal A. (2023). From rejection to the Nobel prize: Karikó and Weissman’s pioneering work on mRNA vaccines, and the need for diversity and inclusion in translational immunology. Front. Immunol. 14:1306025. doi: 10.3389/fimmu.2023.1306025, 38022662 PMC10663363

[ref4] Benedek M. Fink A. (2019). Toward a neurocognitive framework of creative cognition: the role of memory, attention, and cognitive control. Curr. Opin. Behav. Sci. 27, 116–122. doi: 10.1016/j.cobeha.2018.11.002

[ref5] Bjork E. L. Bjork R. A. (2011). “Making things hard on yourself, but in a good way: creating desirable difficulties to enhance learning,” in Psychology and the Real World: Essays Illustrating Fundamental Contributions to Society, (New York: Worth Publishers), 56–64.

[ref6] Börnert M. Wilbert J. (2015). Thinking-Aloud Protocols of Piagetian Tasks: Insights into Problem-Solving Processes of Primary School Students Insights into Learning Disabilities, 12, 19–34.

[ref7] Cohen I. B. Smith G. E. (2002). The Cambridge Companion to Newton. Cambridge, United Kingdom: Cambridge University Press.

[ref8] eds. Davidson J. E. Sternberg R. J. (2003). The Psychology of Problem Solving. Cambridge: Cambridge University Press.

[ref9] Ericsson K. A. (2006). “The influence of experience and deliberate practice on the development of superior expert performance,” in The Cambridge Handbook of Expertise and Expert Performance, (New York: Cambridge University Press), 683–703.

[ref10] Freire P. (1970). The adult literacy process as cultural action for freedom. Harv. Educ. Rev. 40, 205–225. doi: 10.17763/haer.40.2.q7n227021n148p26

[ref11] Funke J. (2010). Complex problem solving: a case for complex cognition? Cogn. Process. 11, 133–142. doi: 10.1007/s10339-009-0345-0, 19902283

[ref12] Gallopoulos E. Houstis E. Rice J. R. (1994). Computer as thinker/doer: problem-solving environments for computational science. IEEE Comput. Sci. Eng. 1, 11–23. doi: 10.1109/99.326669

[ref13] George D. A. S. Baskar D. T. Srikaanth D. P. B. (2024). The erosion of cognitive skills in the technological age: how reliance on technology impacts critical thinking, problem-solving, and creativity. PUIRP 2, 147–163. doi: 10.5281/zenodo.11671150

[ref14] Greene M. T. (1989). Alfred Wegener and the Theory of Continental Drift. NSF Award 88, 22468. Baltimore: Johns Hopkins University Press.

[ref15] Han B. (2020). Next-generation scientists: past, present and future. Innovation 1:100037. doi: 10.1016/j.xinn.2020.100037, 34557711 PMC8454619

[ref16] Kim K. H. (2011). The creativity crisis: the decrease in creative thinking scores on the Torrance tests of creative thinking. Creat. Res. J. 23, 285–295. doi: 10.1080/10400419.2011.627805

[ref17] Kotovsky K. Hayes J. R. Simon H. A. (1985). Why are some problems hard? Evidence from tower of Hanoi. Cogn. Psychol. 17, 248–294. doi: 10.1016/0010-0285(85)90009-X

[ref18] Lu D. Xie Y.-N. (2024). The application of educational technology to develop problem-solving skills: a systematic review. Think. Skills Creat. 51:101454. doi: 10.1016/j.tsc.2023.101454

[ref19] Marshall B. Adams P. C. (2008). *Helicobacter pylori*: A Nobel pursuit? Can. J. Gastroenterol. 22, 895–896. doi: 10.1155/2008/459810, 19018331 PMC2661189

[ref20] Martin M. M. Rubin R. B. (1995). A new measure of cognitive flexibility. Psychol. Rep. 76, 623–626. doi: 10.2466/pr0.1995.76.2.623

[ref21] Meyer R. Desai S. P. (2015). Accepting pain over comfort: resistance to the use of anesthesia in the mid-19th century. J. Anesth. Hist. 1, 115–121. doi: 10.1016/j.janh.2015.07.027, 26828088

[ref22] Miettinen R. (2000). The concept of experiential learning and John Dewey’s theory of reflective thought and action. Int. J. Lifelong Educ. 19, 54–72. doi: 10.1080/026013700293458

[ref23] Park M. Leahey E. Funk R. J. (2023). Papers and patents are becoming less disruptive over time. Nature 613, 138–144. doi: 10.1038/s41586-022-05543-x, 36600070

[ref24] Polya G. (2014). How to Solve It: A New Aspect of Mathematical Method. New Jersey: Princeton University Press.

[ref25] Ramírez-Montoya M. S. Portuguez-Castro M. Mendoza-Urdiales R. (2025). Redefining education: educational trajectories for complex thinking skills. Humanit. Soc. Sci. Commun. 12:958. doi: 10.1057/s41599-025-04929-2

[ref26] Rong Y. Chen N. Dong J. Li Q. Yue X. Hu L. . (2022). Expectations of immediate and delayed reward differentially affect cognitive task performance. NeuroImage 262:119582. doi: 10.1016/j.neuroimage.2022.119582, 35995376

[ref27] Runco M. A. Acar S. (2012). Divergent thinking as an indicator of creative potential. Creat. Res. J. 24, 66–75. doi: 10.1080/10400419.2012.652929

[ref28] Sagan C. (1994). The demon-Haunted World: Science as a Candle in the dark: draft. Available online at: https://www.loc.gov/resource/mss85590.004/ (accessed February 21, 2025).

[ref29] Sahin H. (2021). The effect of STEM-based education program on problem solving skills of five year old children. Malays. Online J. Educ. Technol. 9, 69–88.

[ref30] Samuelson W. Zeckhauser R. (1988). Status quo bias in decision making. J. Risk Uncertain. 1, 7–59. doi: 10.1007/BF00055564

[ref31] Simon H. A. (1973). The structure of ill structured problems. Artif. Intell. 4, 181–201. doi: 10.1016/0004-3702(73)90011-8

[ref32] Simon H. A. Newell A. (1971). Human problem solving: the state of the theory in 1970. Am. Psychol. 26, 145–159. doi: 10.1037/h0030806

[ref33] Stadler M. Herborn K. Mustafić M. Greiff S. (2020). The assessment of collaborative problem solving in PISA 2015: an investigation of the validity of the PISA 2015 CPS tasks. Comput. Educ. 157:103964. doi: 10.1016/j.compedu.2020.103964

[ref34] Thorndike R. L. (1947). Research Problems and Techniques. Washington DC: U.S. Government Printing Office.

[ref35] Tulchinsky T. H. (2018). “John snow, cholera, the broad street pump; waterborne diseases then and now,” in Case Studies in Public Health, (London UK: Academic Press.) 77–99.

[ref36] Tversky A. Kahneman D. (1979). Prospect theory: an analysis of decision under risk Levine’s working paper archive. Available online at: https://ideas.repec.org//p/cla/levarc/7656.html (accessed May 23, 2026).

[ref37] Tversky A. Kahneman D. (1981). The framing of decisions and the psychology of choice. Science 211, 453–458. doi: 10.1126/science.7455683, 7455683

[ref38] Tyagi U. Barwal K. C. (2020). Ignac Semmelweis—father of hand hygiene. Indian J. Surg. 82, 276–277. doi: 10.1007/s12262-020-02386-6, 32837058 PMC7240806

[ref39] Vitello M. Salvi C. (2023). Gestalt’s perspective on insight: a recap based on recent behavioral and neuroscientific evidence. J. Intelligence 11:224. doi: 10.3390/jintelligence11120224, 38132842 PMC10743969

[ref40] Vygotsky L. S. Cole M. (1978). Mind in Society: Development of Higher Psychological Processes. London, UK: Harvard University Press.

[ref41] Weisberg R. Suls J. M. (1973). An information-processing model of Duncker’s candle problem. Cogn. Psychol. 4, 255–276. doi: 10.1016/0010-0285(73)90014-5

[ref42] Wertheimer M. (1938). “The syllogism and productive thinking,” in A Source Book of Gestalt Psychology, (London: Kegan Paul, Trench, Trubner & Company), 274–282.

[ref43] Winkler R. (2006). Does ‘better’ discounting lead to ‘worse’ outcomes in long-run decisions? The dilemma of hyperbolic discounting. Ecol. Econ. 57, 573–582. doi: 10.1016/j.ecolecon.2005.05.013

